# Tolerability and acceptability of electroporation during a Phase 1 vaccine trial at two sites in Uganda and Rwanda

**DOI:** 10.1186/1742-4690-9-S2-P126

**Published:** 2012-09-13

**Authors:** J Mpendo, R Bayingana, A Nanvubya, E Karita, A Ssetaala, N Kiwanuka, J Lehrman, C Schmidt, D Hannaman, S Allen, P Fast

**Affiliations:** 1 UVRI-IAVI HIV Vaccine Program, Entebbe, Uganda; 2 PSF, Kigali, Rwanda; 3 International AIDS Vaccine Initiative, NY, USA; 4 International AIDS Vaccine Initiative, NY, USA; 5 Ichor Medical Systems, CA, USA

## Background

DNA vaccines are weakly immunogenic in humans and one way of increasing their immunogenicity is by administering the vaccines using electroporation (EP). This technique increases the uptake of the DNA into the cell by producing an electrical field which makes the cellular membrane more permeable. In the ongoing trial, we are using the ICHOR TriGrid Delivery system (TDS-IM) to administer bilateral injections of an HIV pDNA vaccine by EP into the medial deltoid muscles.

## Methods

We assessed the acceptability and tolerability of Electroporation among 37 volunteers who received two injections by EP/IM at their first vaccination time point by administering a questionnaire after the procedure. Tolerability was assessed at 4 time points: i) before the vaccine was injected, ii) at the time of electrical stimulation, iii) 10 minutes and iv) 30 minutes after the procedure. The pain was graded as none, light, uncomfortable, intense, severe or very severe.

We also sought their opinion on acceptability of the procedure as a method of vaccine administration.

## Results

Of the 37 volunteers, 45.9% felt no pain at time before vaccine was injected. At time of electrical stimulation, 59.5% felt light pain, 29.7 felt uncomfortable and 10.8% felt intense pain. Ten minutes after the procedure, 56.8% felt light pain, 29.7 felt uncomfortable and 13.5% felt no pain. Thirty minutes after procedure, 70.3% felt light pain, 13.5% felt uncomfortable and 2.7% felt intense pain but this was associated with multiple device applications to the site prior to achieving administration. Majority of the volunteers (94.6%) thought that vaccination by EP would be acceptable if the vaccine protected people from acquiring HIV and contributed to scientific knowledge on vaccine administration.

## Conclusion

Electroporation among African volunteers in this trial is tolerable and acceptable. EP may be considered in future for the administration of HIV vaccines.

